# Endothelial Glycocalyx Layer: A Possible Therapeutic Target for Acute Lung Injury during Lung Resection

**DOI:** 10.1155/2017/5969657

**Published:** 2017-12-20

**Authors:** JiaWan Wang, AnShi Wu, Yan Wu

**Affiliations:** Anaesthesia Department of Beijing Chaoyang Hospital Affiliated to Capital Medical University, Beijing, China

## Abstract

**Background:**

Shedding of the endothelial glycocalyx layer (EGL) is known to occur during major surgery, but its degradation associated with minimally invasive video-assisted thoracoscopy (VATS) remains unclear. We investigated if serum biomarkers of EGL disruption were elevated during VATS lobectomy, and whether the urinary trypsin inhibitor (UTI) ulinastatin exerted a protective effect during this procedure.

**Materials and Methods:**

Sixty ASA II-III lung cancer patients undergoing elective VATS lobectomy were divided equally into UTI and control groups. UTI group patients received intravenous UTI during surgery. Serum levels of syndecan-1 and heparan sulfate were examined before (T0) and at the end of surgery (T1). Serum albumin and hemoglobin were measured before surgery (BOD) and on the first postoperative day (POD1).

**Results:**

In control group, syndecan-1 levels were significantly elevated at T1 compared with T0 (3.77 ± 3.15 versus 4.28 ± 3.30, *P* = 0.022^⁎^) and increased even more significantly in patients whose surgery lasted >3 h (3.28 ± 2.84 versus 4.31 ± 3.39, *P* = 0.003^⁎⁎^). Serum albumin levels on POD1 were significantly lower in control group compared with UTI group (32.63 ± 4.57 versus 35.76 ± 2.99, *P* = 0.031^⁎^).

**Conclusion:**

EGL degradation occurs following VATS lobectomy. UTI can alleviate this shedding, thus helping preserve normal vascular permeability.

**Trail Registration:**

This trial is registered with ChiCTR-IOC-17010416 (January 13, 2017).

## 1. Introduction

Lung resection is the preferred therapeutic choice for suitable lung cancer cases. However, acute lung injury (ALI) and induced acute respiratory distress syndrome (ARDS) are the leading cause of death after thoracic surgery, leading to significantly reduced 1-year survival [1–3]. Minimally invasive techniques, such as video-assisted thoracoscopy (VATS) procedures, are thus being increasingly used for lung resection. However, although VATS is less damaging, one-lung ventilation (OLV), which is required for most of the procedures, can also contribute to the development of ALI [4]. ALI and ARDS are parts of the same disease spectrum [5, 6], which includes pulmonary edema as the intrinsic pathologic process; acute lung injury after pulmonary resection has thus been variously described as post-lung resection pulmonary edema, postpneumonectomy pulmonary edema, and low pressure or noncardiogenic pulmonary edema [7, 8].

The endothelial glycocalyx layer (EGL) is a dynamic layer mainly consisting of glycoprotein and proteoglycan layers at the luminal surface of the vascular endothelium. Although the precise mechanism is not well understood, this endothelial surface layer is believed to act as a barrier to circulating cells and large molecules involved in fluid homeostasis and regulation, and it thus plays as key role in vascular permeability and edema formation [7, 9–13]. In light of this new paradigm regarding the relationship between the glycocalyx and lung injury, loss of glycocalyx integrity and damage to the vascular endothelium as a result of ischemia and inflammatory responses caused by surgery and OLV may be major mediators of edema formation leading to ALI. Human neutrophils have been implicated as mediators of tissue-destructive events in ARDS. Neutrophil elastase, a strong serine protease, plays a crucial role in neutrophil-mediated endothelial cell injury, thus contributing to vascular injury [14–16]. Serine protease inhibitors, such as the urinary trypsin inhibitor (UTI) ulinastatin, inhibit neutrophil elastase activity and trypsin activity, with potentially protective effects against organ injury, and are now applied clinically [11, 17]. In this clinical study, we aimed to determine if minimally invasive VATS lobectomy under OLV caused glycocalyx degradation. We also investigated the effect of UTI administration on the concentrations of serum biomarkers of EGL disruption, with implications for the prevention of ALI.

## 2. Methods

### 2.1. Study Design and Procedure

Ethical approval for this clinical cohort study (number 2016-sci-122) was provided by the Ethics Committee of Beijing Chaoyang Hospital Affiliated to Capital Medical University, Beijing, China, and the study was registered at clinicaltrialecrf.org (ChiCTR-IOC-17010416). Sixty ASA II-III lung cancer patients undergoing elective VATS lobectomy were selected to participate in the study. Informed consent was obtained from all patients. Exclusion criteria were body mass index >35 kg/m^2^, cardiac failure (New York Heart Association class >II), pulmonary hypertension (systolic pulmonary arterial pressure >50 mmHg), clinically relevant obstructive or restrictive lung diseases, obvious coagulation disorders, severe pulmonary or systemic infections, treatment with immunodepressant drugs in the 8 weeks before surgery, and tobacco abuse.

The patients were divided randomly into a control group (*n* = 30) and UTI group (*n* = 30) based on a list of random numbers generated by the RAND function in MS Excel. General anesthesia was induced with midazolam 0.02 mg/kg, propofol 1.5–2.5 mg/kg, and sufentanil 0.5 *μ*g/kg. Tracheal intubation using a double-lumen endotracheal tube was facilitated by administration of rocuronium (0.8 mg/kg). The correct position of the tube was confirmed by fiberoptic bronchoscopy. Anesthesia was maintained by continuous infusion of propofol (3–5 mg/kg/h) and remifentanil (0.1–0.4 *μ*g/kg/min), and administration of rocuronium. Supplemental sevoflurane 0.5–1 v/v% was used if needed. The patients were placed in the lateral decubitus position for VATS lobectomy with OLV. The tidal volume was set to 6 ml/kg, the fraction of inspired oxygen (FiO_2_) was adjusted to maintain oxyhemoglobin saturation >95%, and the respiratory rate was set to maintain PaCO_2_ between 35 and 45 mmHg. When the main procedure was finished, double-lung ventilation was restored for reconstruction. Patients in the UTI group received intravenous ulinastatin for injection (UTI; Techpool, Guangdong, China) with a loading dose of 5000 IU/kg at the beginning of surgery, followed by infusion of 1000 IU/kg/h during surgery. The control group was given the same volume of normal saline solution. Lactated Ringer's solution and 6% 130/0.4 hydroxyethyl starch were infused during the procedure, but no exogenous albumin solution was applied.

### 2.2. Samples and Examinations

Blood samples were collected from the radial artery before surgery (T0) and at the end of surgery (T1). The blood samples were centrifuged after placed stable for 1 hour and the serum was then stored at −80°C for future use. The levels of the glycocalyx components syndecan-1 (RayBiotech, Georgia, USA) and HS (Cusabio, Wuhan, China) were measured by enzyme-linked immunosorbent assay (ELISA). Arterial blood gases were analyzed and the PaO_2_/FiO_2_ ratios were calculated at T0 and T1. Plasma albumin and hemoglobin (HGB) levels, fibrinogen degradation products (FDP), fibrinogen (Fbg), D-dimer, and international normalized ratio (INR) were measured before surgery (BOD) and on the first postoperative day (POD1).

### 2.3. Statistical Analysis

Normally distributed data are presented as mean ± standard error of the mean. The results of statistical tests were considered significant if *P* < 0.05. Mean values were compared between groups by independent sample *t*-tests, and mean values within groups were compared by paired* t*-tests. Numerical data were compared by Pearson's *χ*^2^ tests. SPSS 15 and GraphPad Prism software (version 6) were used for statistical analysis and for creating the graphs, respectively.

## 3. Results

**Figure 1:**
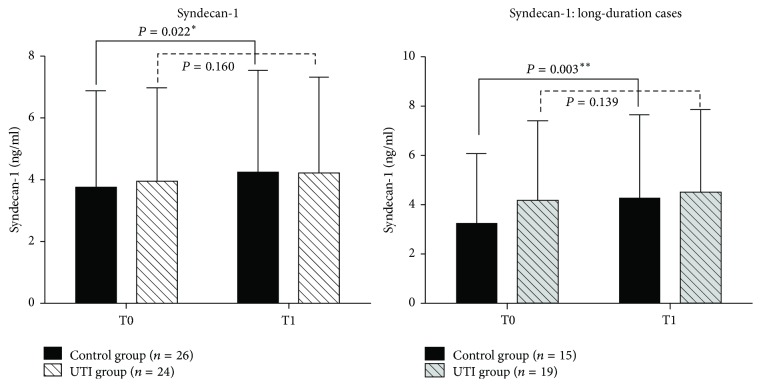
Syndecan-1 levels were significantly elevated in the control group at T1 compared with T0 (3.77 ± 3.15 versus 4.28 ± 3.30, *P* = 0.022^*∗*^), whereas the increase was not significant in the UTI group (3.98 ± 3.04 versus 4.24 ± 3.12, *P* = 0.160). The procedure lasted >3 h in 15 patients in the control group and 19 in the UTI group (long-duration cases). Syndecan-1 levels were elevated more significantly at T1 compared with T0 among long-duration cases (3.28 ± 2.84 versus 4.31 ± 3.39, *P* = 0.003^*∗∗*^), whereas the increase was still not significant in the equivalent UTI group (4.17 ± 3.29 versus 4.51 ± 3.41, *P* = 0.138).

**Figure 2:**
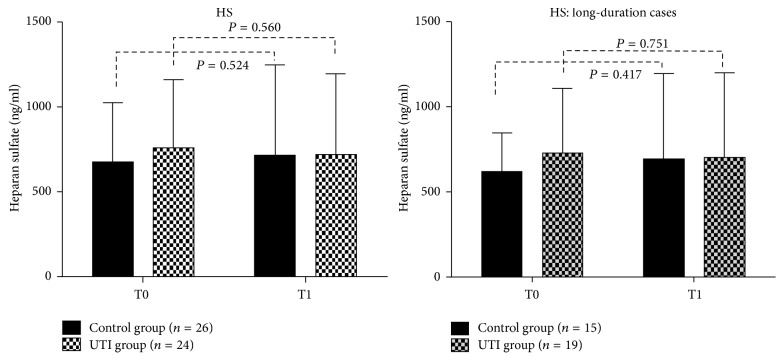
There was no obvious variation in HS levels in either group overall or among long-duration cases.

**Figure 3:**
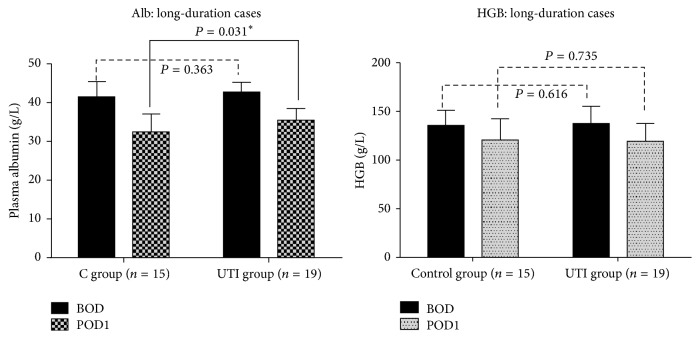
Serum albumin levels were significantly lower in the control compared with the UTI long-duration group at POD1 (32.63 ± 4.57 versus 35.76 ± 2.99, *P* = 0.031^*∗*^), while there was no significant difference in HGB levels between the two groups.

Twenty-eight patients in the control group and 27 in the UTI group received a definite pathologic diagnosis of lung cancer. In these patients, blood sample hemolysis happened in two patients in the control group and three patients in the UTI group which were rejected. So finally twenty-six patients in the control group and 24 in the UTI group were enrolled in the study. There were no significant differences between the UTI and control groups in terms of baseline characteristics, duration of operation, perioperative blood loss, fluid volume, urine volume, or duration of hospital stay (Table 1). HGB and serum albumin levels were also similar in the two groups at BOD. However, syndecan-1 levels were significantly elevated at T1 compared with T0 in the control group (3.77 ± 3.15 versus 4.28 ± 3.30, *P* = 0.022), whereas the increase in syndecan-1 levels was not significant in the UTI group (3.98 ± 3.04 versus 4.24 ± 3.12, *P* = 0.160). The procedure lasted >3 h in 15 control and 19 UTI cases (long-duration groups). Further analysis of these long-duration cases showed that syndecan-1 levels were highly significantly elevated at T1 compared with T0 in the control group (3.28 ± 2.84 versus 4.31 ± 3.39, *P* = 0.003), whereas the increase was still not significant in the UTI group (4.17 ± 3.29 versus 4.51 ± 3.41, *P* = 0.138) (Figure 1). There were no obvious variations in HS in either group (Figure 2). Serum albumin and HGB were significantly reduced at POD1 compared with BOD among the long-duration cases (Table 2), and POD1 serum albumin levels were significantly lower in the control group compared with the UTI group (32.63 ± 4.57 versus 35.76 ± 2.99, *P* = 0.031). There was no significant difference in HGB levels between the two groups over the same time periods (Figure 3), and there were no obvious variations in PO_2_/FiO_2_ or plasma lactic acid levels in either group (Table 3). Fbg, FDP, D-dimer, and INR increased significantly at POD1 compared with BOD in both groups (Table 4), but there was no significant difference in any of these parameters between the two groups at either BOD or POD1 (Table 5).

## 4. Discussion

In this study, we treated patients undergoing VATS lobectomy with the UTI ulinastatin at the routine dose permitted in China, as used in other studies [18, 19]. Serum syndecan-1 levels in patients without UTI treatment increased significantly by nearly 20% following VATS lobectomy, with an even greater elevation of about 30% in cases whose operation lasted >3 h. These results indicated that VATS lobectomy resulted in direct and duration-related shedding of the EGL. In contrast, there was no significant elevation in serum syndecan-1 levels in the UTI group, even among patients with operation times >3 h, indicating that UTI helped to preserve the EGL during the procedure. There was no significant difference in plasma albumin or HGB levels between the control and UTI groups at BOD, though plasma albumin levels, but not HGB levels, were significantly lower in the control group at POD1 (18 h after surgery) compared with the UTI group. This suggests that there was more perioperative transcapillary albumin leakage in the control group. The abovementioned decrease in HGB was likely due not only to blood loss, but also to dilution caused by the infusion of about 2 l of crystalloid and colloid solutions. There was no significant increase in heparan sulfate (HS) levels in either group at the end of surgery compared with those at baseline.

Deterioration of the EGL is known to be caused by major surgery, and serum syndecan-1 and HS are important biomarkers of this process [20–24]. Different types of surgery have been associated with perioperative variations in serum syndecan-1 and HS, but the details have differed. In a study of 28 patients undergoing major abdominal surgery, Steppan et al. found that syndecan-1 levels peaked 24 h postoperatively, with around a 1.5-fold increase compared with baseline, but there was no significant perioperative elevation in HS levels [20]. Rehm et al. investigated patients who underwent abdominal aortic aneurysm repair without cardiopulmonary bypass and who had infrarenal total ischemia and found a 15-fold increase in the median syndecan-1 concentration 15 min after declamping and a slow 2-fold increase in HS after surgery. There was also a selective 2.5-fold rise in HS levels 24 h after surgery, but syndecan-1 shedding remained unchanged compared with the preoperative level [21]. Svennevig et al. measured syndecan-1 levels in 44 patients undergoing coronary artery bypass grafting, both with and without cardiopulmonary bypass, and found that syndecan-1 levels peaked in both groups at the end of the operation, with a 4-fold elevation by POD1 [22]. In a pilot study including patients over 16 years undergoing elective open lobectomy for primary lung cancer, Arthur et al. reported no significant change in HS levels and a delayed elevation of about 50% in syndecan-1 levels at POD2 [23]. Deterioration of the EGL following major surgery has also been detected in infants [24]. However, these previous studies lacked intraoperative data for lung resection, and we therefore detected changes in syndecan-1 and HS levels near the end of surgery. It is assumed that minor disturbances to the EGL that only result in damage to the most luminal component would result in isolated elevation of HS, while more significant damage resulting in degradation of the glycoprotein backbone adherent to the endothelial cell surface would cause elevation of syndecan-1 [21]; however, in accord with Steppan et al. and Arthur et al., we found no significant change in HS levels in the current study. This apparent discrepancy may arise because of rapid renal clearance of HS [25, 26]. Furthermore, the use of different ELISA products may have contributed to differences among the studies in terms of the levels of syndecan-1 and HS.

The function of the EGL in the setting of VATS lobectomy has not previously been explored. The thickness of the endothelial surface layer in pulmonary vessels is considered to exceed that in other vascular beds, indicating an important barrier function in lung vessels [27]. It is therefore reasonable to imagine that lung surgery may more easily cause increases in circulating syndecan-1 and HS, as degradation components of the endothelial surface layer. Although VATS is minimally invasive [28], the lobectomy procedure still causes damage that may result in the observed effect on EGL. Preoperatively, cancer patients have a higher oxidative burden and reduced antioxidant capacity, and their tissues will thus be less tolerant to ischemia−reperfusion injury [29]. Intraoperatively, surgical manipulation, deflation with the potential for traumatic shearing of the EGL, tissue ischemia, and reperfusion injury are all well-known independent factors in EGL degradation. Furthermore, OLV associated with volutrauma, atelectrauma, hyperperfusion/capillary shear stress, and oxidative injury following prolonged exposure to high FiO_2_ may also contribute to EGL disruption [7, 30]. Glycocalyx damage impairs a number of important endothelial cell functions leading to increased microvascular permeability and leakage of fluid and plasma proteins into the interstitium, with resultant tissue edema [31]. All the cases in the current study showed significantly greater decreases in plasma albumin compared with HGB at POD1, suggesting that serum albumin was lost not only as a result of blood loss, but also by extravasation attributed to increased vascular permeability [32]. Furthermore, serum albumin levels were significantly lower at POD1 in the control group compared with the UTI group, while HGB levels were similar in both groups. This also suggests that plasma albumin leakage was reduced as a result of the decrease in microvascular permeability in the UTI group. UTI is an endogenous acidic glycoprotein secreted by the liver that can be purified from fresh urine from healthy men. It suppresses a wide spectrum of serine proteases and excessive expression of proinflammatory cytokines [33, 34]. Serine proteases include trypsin, thrombin, chymotrypsin, kallikrein, plasmin, neutrophil elastase, cathepsin, neutrophil protease-3, and the coagulation factors IXa, Xa, XIa, and XlIa, which play important roles in the regulation of inflammation via inter- and intracellular signaling pathways [35, 36]. Neutrophils are generally recognized to play central roles in the pathogenesis of ALI secondary to mechanical insults [37], while neutrophil elastase is involved in endothelial cell injury mediated by stimulated neutrophils. UTI suppresses the production and secretion of activated elastase by neutrophils and inactivates secreted extracellular elastase, as well as rescuing the endogenous A1 protease inhibitor [17, 38]. In the present study, UTI decreased the circulatory levels of EGL-degradation components towards the end of surgery and reduced plasma albumin leakage at POD1, implying that UTI protected the endothelial surface layer during VATS lobectomy. This may represent the mechanism whereby UTI preserves vascular endothelial barrier function and mitigates tissue edema. However, we found no significant effects of UTI on fibrinolysis indicators such as FDP, Fbg, and D-dimer, which can be associated with circulatory EGL function [39], and further studies are needed to investigate these relationships, possibly using a larger UTI dose. We also found no variations in PO_2_/FiO_2_ and lactic acid, though this may have been because the sample size was not powerful enough to investigate the relationships between biomarkers of EGL shedding and clinical outcomes such as poor tissue oxygenation. It is also possible that our results were affected by not using a unified stepwise method to analyze lung recruitment [40], and this should be investigated in future studies. Another limitation of the study was the use of only one dose of UTI; given that UTI inhibits intracellular elastase activity in a dose-dependent fashion, further studies should be carried out using different UTI doses.

## 5. Conclusion

ALI after lung resection remains a poorly understood condition with high mortality and limited therapeutic options. EGL dysfunction has demonstrated a close relationship with ALI and ARDS. This study demonstrated EGL shedding following VATS lobectomy and confirmed that the applied dose of UTI could alleviate this degradation and decrease albumin extravasation, thus preserving vascular permeability. These results suggest that the glycocalyx may represent a therapeutic target for improving ALI following lung resection. Further studies are required to investigate EGL function in the perioperative setting and to explore the effects of different UTI doses on the EGL and clinical outcomes.

## Figures and Tables

**Table 1:** Patient characteristics and basic operative data.

	Control group** (***n* = 26**)**	UTI group** (***n* = 24**)**	*P*
Men/women	11/15	11/13	0.802
Age (years)	58.73 ± 8.58	58.38 ± 10.24	0.894
Bodyweight (kg)	63.00 ± 11.39	64.58 ± 8.53	0.583
Height (cm)	161.88 ± 7.51	164.79 ± 7.99	0.191
Operation time (min)	192.31 ± 69.17	187.92 ± 48.61	0.798
Crystalloid solution (ml)	1180.76 ± 452.57	1308.33 ± 492.48	0.345
Colloid solution (ml)	750.00 ± 353.55	729.17 ± 254.49	0.813
Red blood cell (ml)	0.00 ± 0.00	16.67 ± 81.65	0.303
Plasma (ml)	0.00 ± 0.00	16.67 ± 81.65	0.303
Blood lost (ml)	182.69 ± 165.49	219.58 ± 166.90	0.437
Urine volume (ml)	526.92 ± 301.74	422.92 ± 167.45	0.143
Hospital stay (days)	6.65 ± 3.55	6.70 ± 4.27	0.970

Data presented as mean ± standard deviation or number.

**Table 2:** Intragroup comparison of serum albumin and HGB levels in long-duration cases.

		BOD	POD1	*P*
Plasma albumin (g/L)	Control group (*n* = 15)	41.74 ± 3.82	32.63 ± 4.57	0.000^*∗∗*^
	UTI group (*n* = 19)	42.89 ± 2.61	35.76 ± 2.99	0.000^*∗∗*^
HGB (g/L)	Control group (*n* = 15)	136.53 ± 15.92	121.47 ± 21.65	0.002^*∗∗*^
	UTI group (*n* = 19)	138.53 ± 18.04	120.42 ± 18.43	0.000^*∗∗*^

Data presented as mean ± standard deviation, ^*∗∗*^*P* < 0.01.

**Table 3:** Intergroup comparison of PO_2_/FiO_2_ and lactic acid levels.

		Control group	UTI group	*P*
PO_2_/FiO_2_	T0	232.55 ± 195.33	322.38 ± 144.30	0.168
	T1	336.00 ± 94.97	388.40 ± 138.78	0.244
Lac (mmol/L)	T0	1.41 ± 0.51	1.11 ± 0.44	0.099
	T1	1.34 ± 0.38	1.05 ± 0.41	0.057

Data presented as mean ± standard deviation.

**Table 4:** Intragroup comparison of FDP, Fbg, D-dimer, and INR.

		BOD	POD1	*P*
FDP (*μ*g/ml)	Control group (*n* = 26)	1.76 ± 1.11	6.87 ± 5.19	0.000^*∗∗*^
	UTI group (*n* = 24)	1.84 ± 1.70	5.04 ± 2.63	0.000^*∗∗*^
Fbg (mg/dl)	Control group (*n* = 26)	307.30 ± 118.49	329.64 ± 125.57	0.286
	UTI group (*n* = 24)	278.15 ± 78.01	294.70 ± 80.56	0.215
D-dimer (mg/L)	Control group (*n* = 26)	0.36 ± 0.42	2.30 ± 1.96	0.000^*∗∗*^
	UTI group (*n* = 24)	0.37 ± 0.64	1.57 ± 1.08	0.000^*∗∗*^
INR	Control group (*n* = 26)	1.02 ± 0.08	1.08 ± 0.11	0.000^*∗∗*^
	UTI group (*n* = 24)	1.01 ± 0.67	1.11 ± 0.08	0.000^*∗∗*^

Data presented as mean ± standard deviation, ^*∗∗*^*P* < 0.01.

**Table 5:** Intergroup comparison of FDP, Fbg, D-dimer, and INR between control and UTI groups.

		Control group (*n* = 26)	UTI group (*n* = 24)	*P*
FDP (*μ*g/ml)	BOD	1.76 ± 1.11	1.84 ± 1.70	0.854
	POD1	6.87 ± 5.19	5.04 ± 2.63	0.142
Fbg (mg/dl)	BOD	307.30 ± 118.49	278.15 ± 78.01	0.314
	POD1	329.64 ± 125.57	294.70 ± 80.56	0.252
D-dimer (mg/L)	BOD	0.36 ± 0.42	0.37 ± 0.64	0.963
	POD1	2.30 ± 1.96	1.57 ± 1.08	0.128
INR	BOD	1.02 ± 0.08	1.01 ± 0.67	0.778
	POD1	1.08 ± 0.11	1.11 ± 0.08	0.290

Data presented as mean ± standard deviation.
